# Measurement of Cannabis Consumption to Determine Risk and Promote Public Health

**DOI:** 10.1192/j.eurpsy.2023.85

**Published:** 2023-07-19

**Authors:** A. J. Budney

**Affiliations:** Psychiatry and Biomedical Data Sciences, Geisel School of Medicine at Dartmouth, Lebanon, United States

## Abstract

**Abstract:**

With the escalation of cannabis legalization and commercialization, the need to differentiate low- vs. high-risk patterns of cannabis use, especially among frequent consumers, becomes essential for development of prevention and intervention strategies and public health messaging. The diversity of cannabis products and methods of intake make this task complex. In particular, the lack of valid methods for quantifying use of the intoxicating component of cannabis, i.e., THC, poses a difficult challenge. This presentation will describe a series of internet-based, personalized survey studies of adults who consume cannabis frequently. The aims of the studies are to develop methods for quantifying THC from self-reports of use, identify patterns of use, and determine associations between use and risk. In the first study of adult daily cannabis consumers (n>4000), rates of CUD were 35% no disorder, 39% mild, 18% moderate, 8% severe disorder. Higher severity was significantly related to younger age, unemployment, and specific reasons for use. Latent class analyses identified four distinct subgroups and preliminary analyses showed that those more likely to report oral use were less likely to meet CUD criteria, and those more likely to report use of high potency products were more likely to meet moderate/severe criteria. Two studies (n’s >2000) compared different quantitative formulas for estimating daily THC consumption from vaping or smoking cannabis products. Findings demonstrated how quantity (mgTHC) relates to socio-demographics, use patterns, and CUD severity. However, substantial variability in the estimates obtained across quantitation methods indicates the need for additional studies to determine optimal approaches. Overall, findings show that specific characteristics of use can discriminate low- from high-risk consumption among those who use frequently, which is critical for developing cannabis policy and public health messaging.

**Disclosure of Interest:**

None Declared

